# Supplementary material for: A Method for Increasing the Robustness of Stable Feature Selection for Biomarker Discovery in Molecular Medicine Developed Using Serum Small Extracellular Vesicle Associated miRNAs and the Barrett’s Oesophagus Disease Spectrum
Source: Int J Mol Sci. 2023 Apr 11;24(8):7068. doi: 10.3390/ijms24087068 (PMC10139127; doi:10.3390/ijms24087068)
Supplement: Supplementary file 1 [file ijms-24-07068-s001.zip › ijms-2304691-supplementary.pdf]

Supplementary Data.

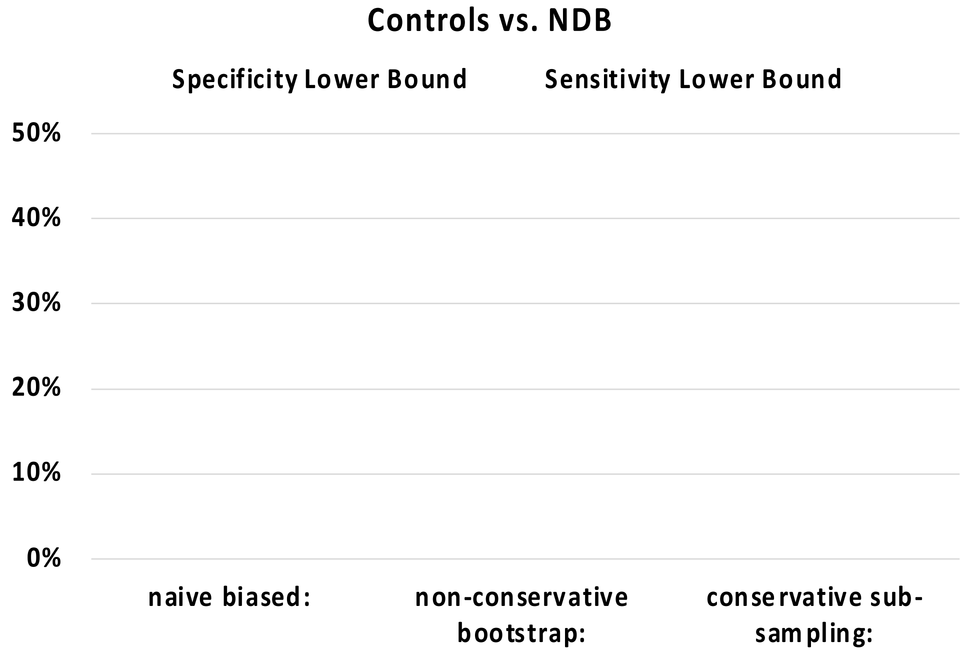

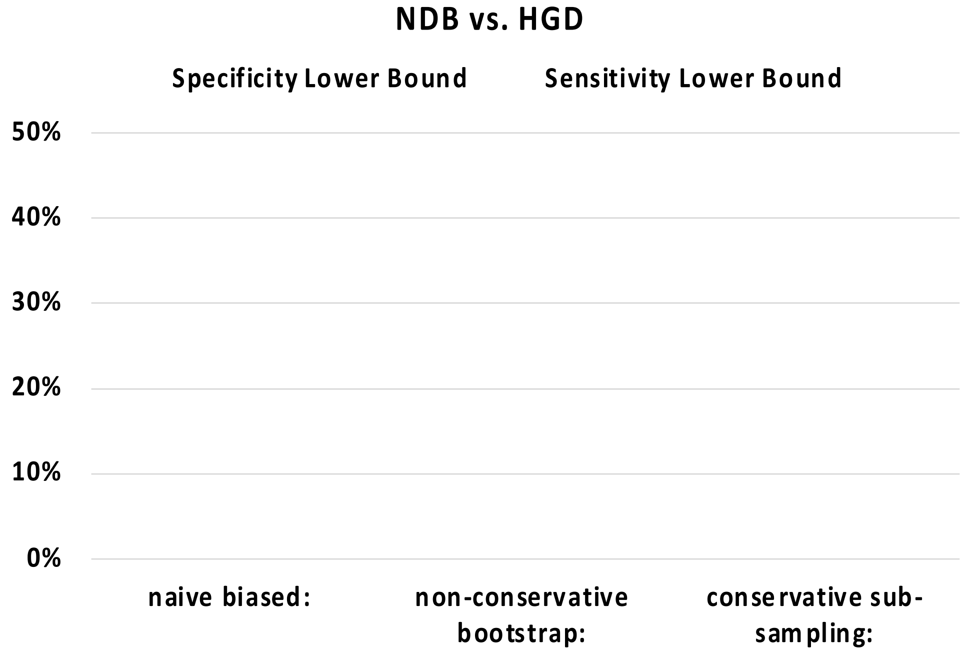

Figure S1. Standard nested cross validation: naive biased, non-conservative bootstrap, and *conservative* sub-sampling per sample derived prediction probability 95% confidence interval lower bounds for the estimates of specificity and sensitivity. **Blue bars** are 95% confidence interval lower bounds on the Specificity, and **red bars** are 95% confidence interval lower bounds on the Sensitivity.

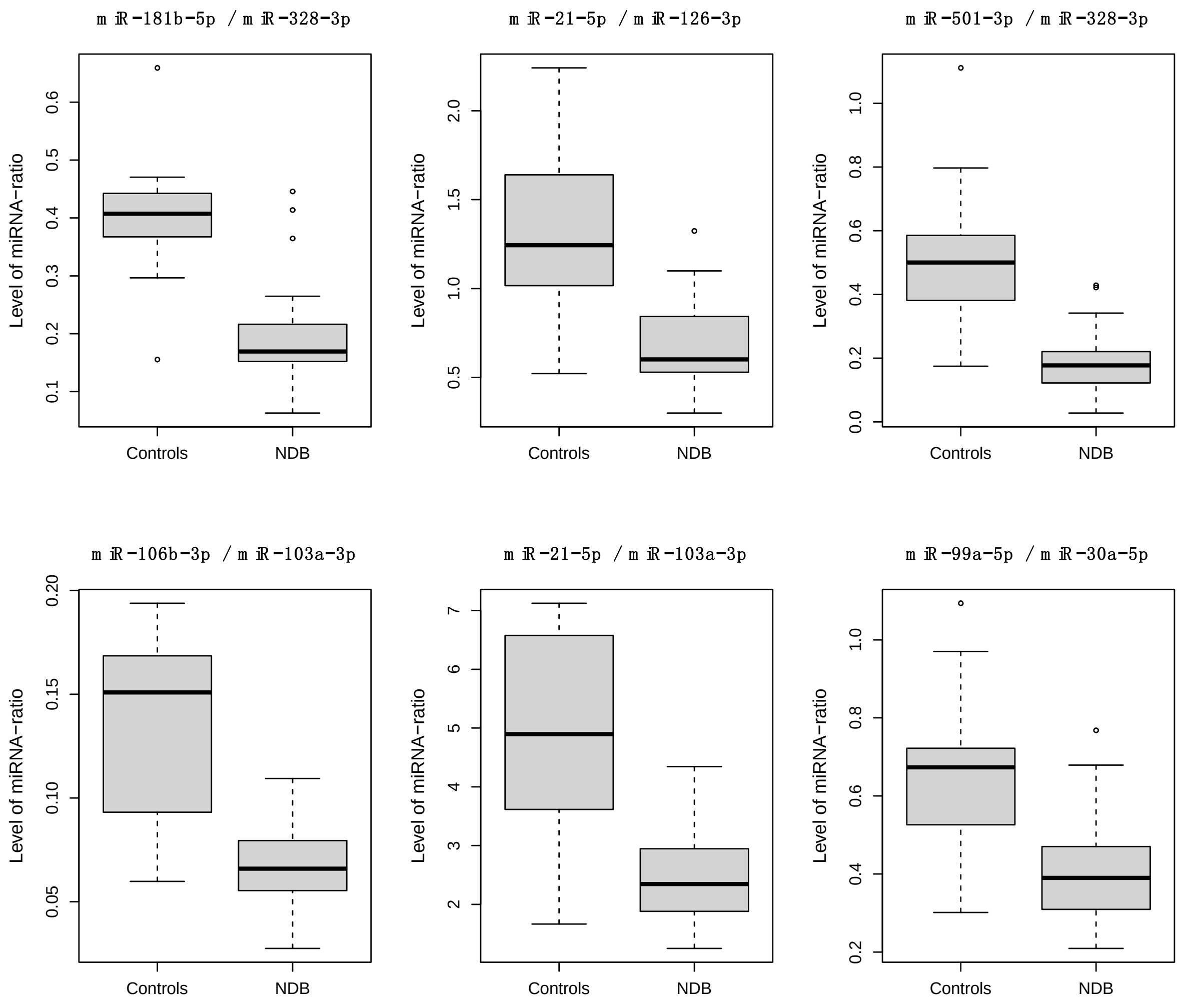

Figure S2. Controls vs. NDB: boxplots for miRNA-ratios in final models.
Box (25th – 75th percentiles) and whisker (5th and 95th percentiles) plots with median values shown by a thick black line. Outlier values are shown by small open circles.

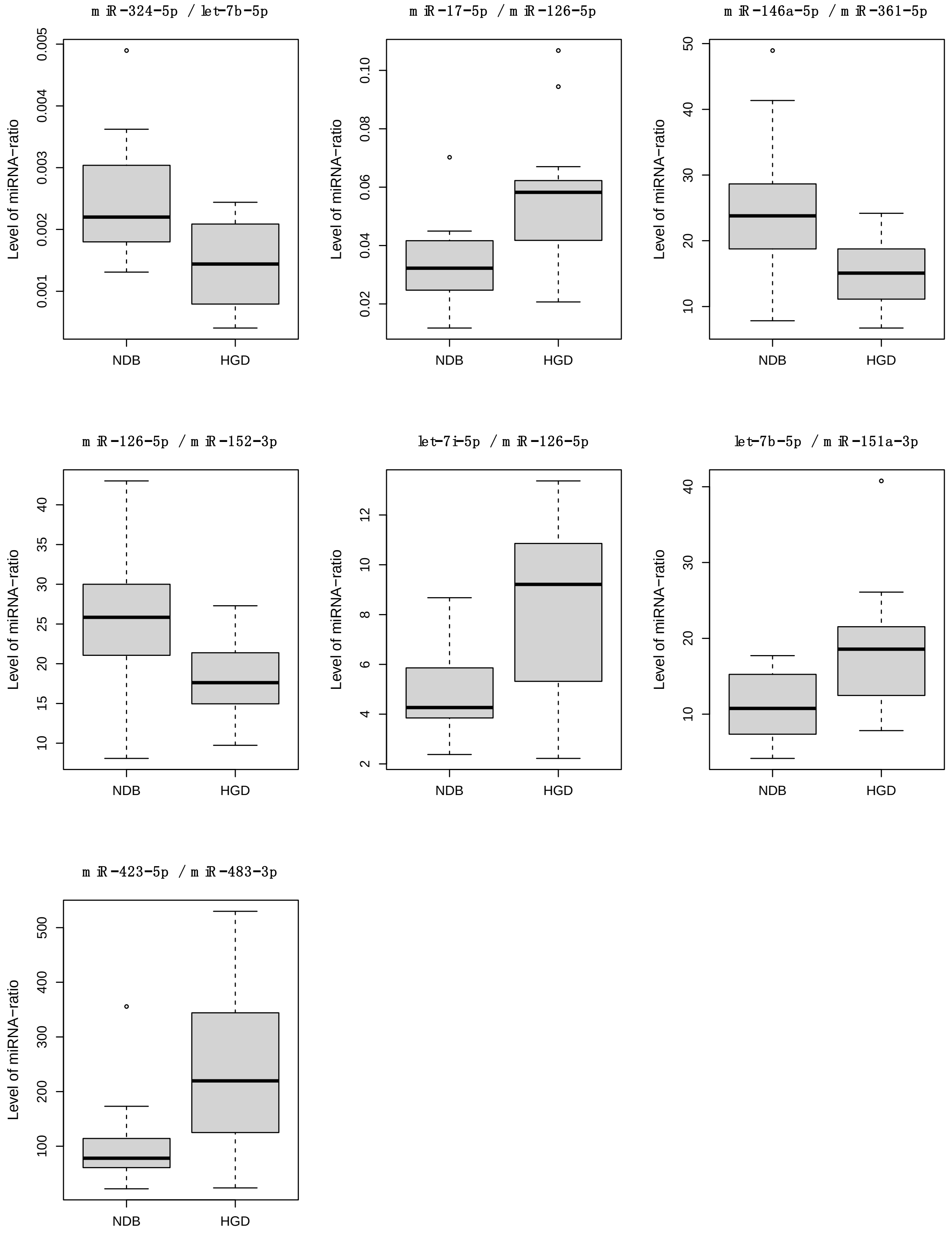

Figure S3. NDB vs. HGD: boxplots for miRNA-ratios in final models.
Box (25th – 75th percentiles) and whisker (5th and 95th percentiles) plots with median values shown by a thick black line. Outlier values are shown by small open circles.

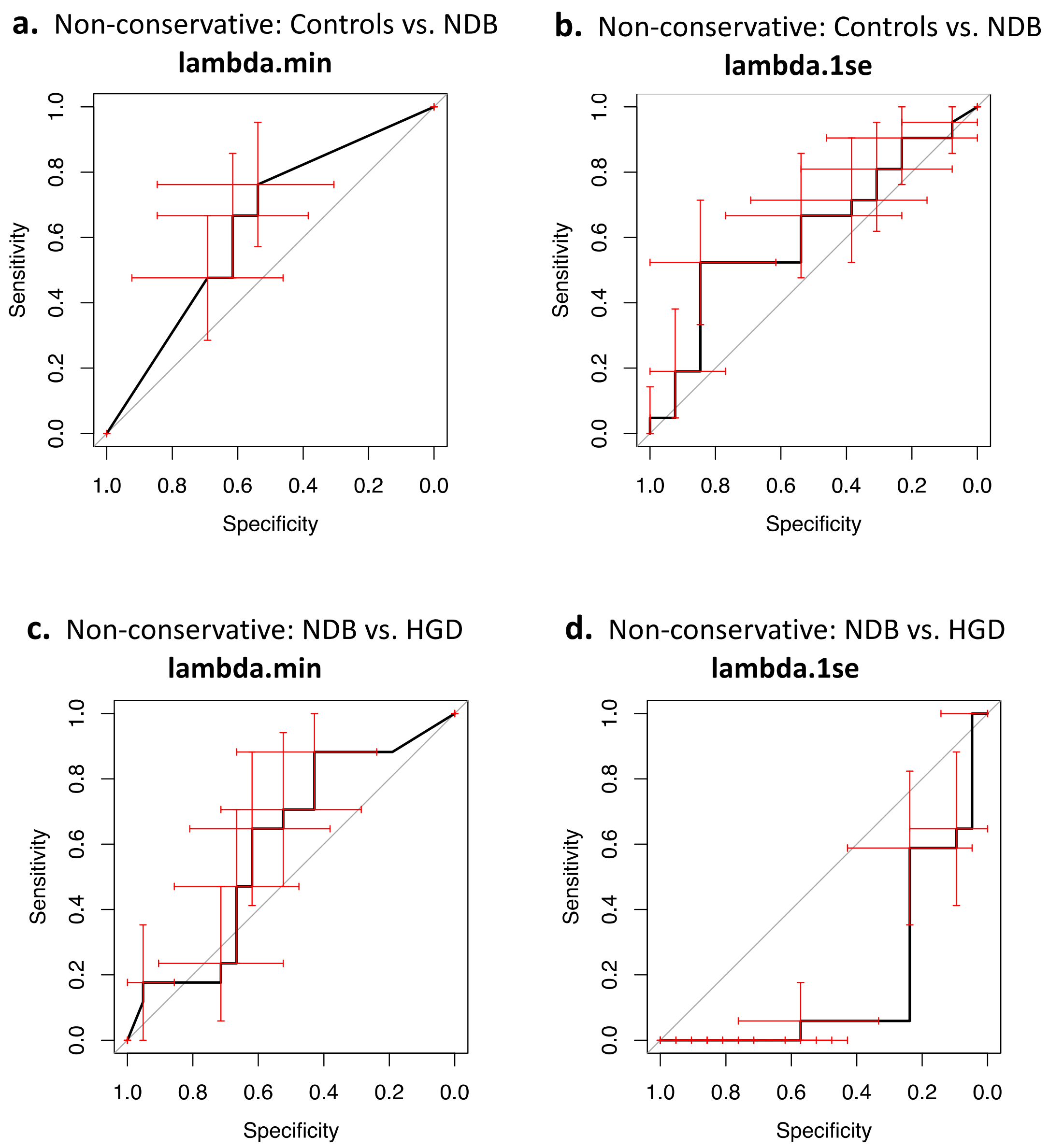

Figure S4. Effects of increased regularisation on predictive capacity.
Lambda.min refers to the optimum level of penalisation for LASSO regression that minimises the prediction error. **Lambda.1se** refers to an increased level of penalisation for LASSO regression that lies within one standard error of lambda.min. ROC curve error bars (red) are non-conservative bootstrapped 95% confidence intervals.

Supplementary Table S1. Details of miRNAs in final model miRNA-ratios for Controls vs. NDB.

	miRBase v22 ID	miRBase v22 mature sequence	miRbase Accession
1	hsa-miR-181b-5p	AACAUUCAUUGCUGUCGGUGGGU	MIMAT0000257
2	hsa-miR-328-3p	CUGGCCCUCUCUGCCCUUCCGU	MIMAT0000752
3	hsa-miR-21-5p	UAGCUUAUCAGACUGAUGUUGA	MIMAT0000076
4	hsa-miR-126-3p	UCGUACCGUGAGUAAUAAUGCG	MIMAT0000445
5	hsa-miR-501-3p	AAUGCACCCGGGCAAGGAUUCU	MIMAT0004774
6	hsa-miR-106b-3p	CCGCACUGUGGGUACUUGCUGC	MIMAT0004672
7	hsa-miR-103a-3p	AGCAGCAUUGUACAGGGCUAUGA	MIMAT0000101
8	hsa-miR-99a-5p	AACCCGUAGAUCCGAUCUUGUG	MIMAT0000097
9	hsa-miR-30a-5p	UGUAAACAUCCUCGACUGGAAG	MIMAT0000087

Supplementary Table S2. Details of miRNAs in final model miRNA-ratios for NDB vs. HGD.

	miRBase v22 ID	miRBase v22 mature sequence	miRbase Accession
1	hsa-miR-324-5p	CGCAUCCCCUAGGGCAUUGGUG	MIMAT0000761
2	hsa-let-7b-5p	UGAGGUAGUAGGUUGUGUGGUU	MIMAT0000063
3	hsa-miR-17-5p	CAAAGUGCUUACAGUGCAGGUAG	MIMAT0000070
4	hsa-miR-126-5p	CAUUAUUACUUUUGGUACGCG	MIMAT0000444
5	hsa-miR-146a-5p	UGAGAACUGAAUUCCAUGGGUU	MIMAT0000449
6	hsa-miR-361-5p	UUAUCAGAAUCUCCAGGGGUAC	MIMAT0000703
7	hsa-miR-152-3p	UCAGUGCAUGACAGAACUUGG	MIMAT0000438
8	hsa-let-7i-5p	UGAGGUAGUAGUUUGUGCUGUU	MIMAT0000415
9	hsa-miR-151a-3p	CUAGACUGAAGCUCCUUGAGG	MIMAT0000757
10	hsa-miR-423-5p	UGAGGGGCAGAGAGCGAGACUUU	MIMAT0004748
11	hsa-miR-483-3p	UCACUCCUCUCCUCCCGUCUU	MIMAT0002173

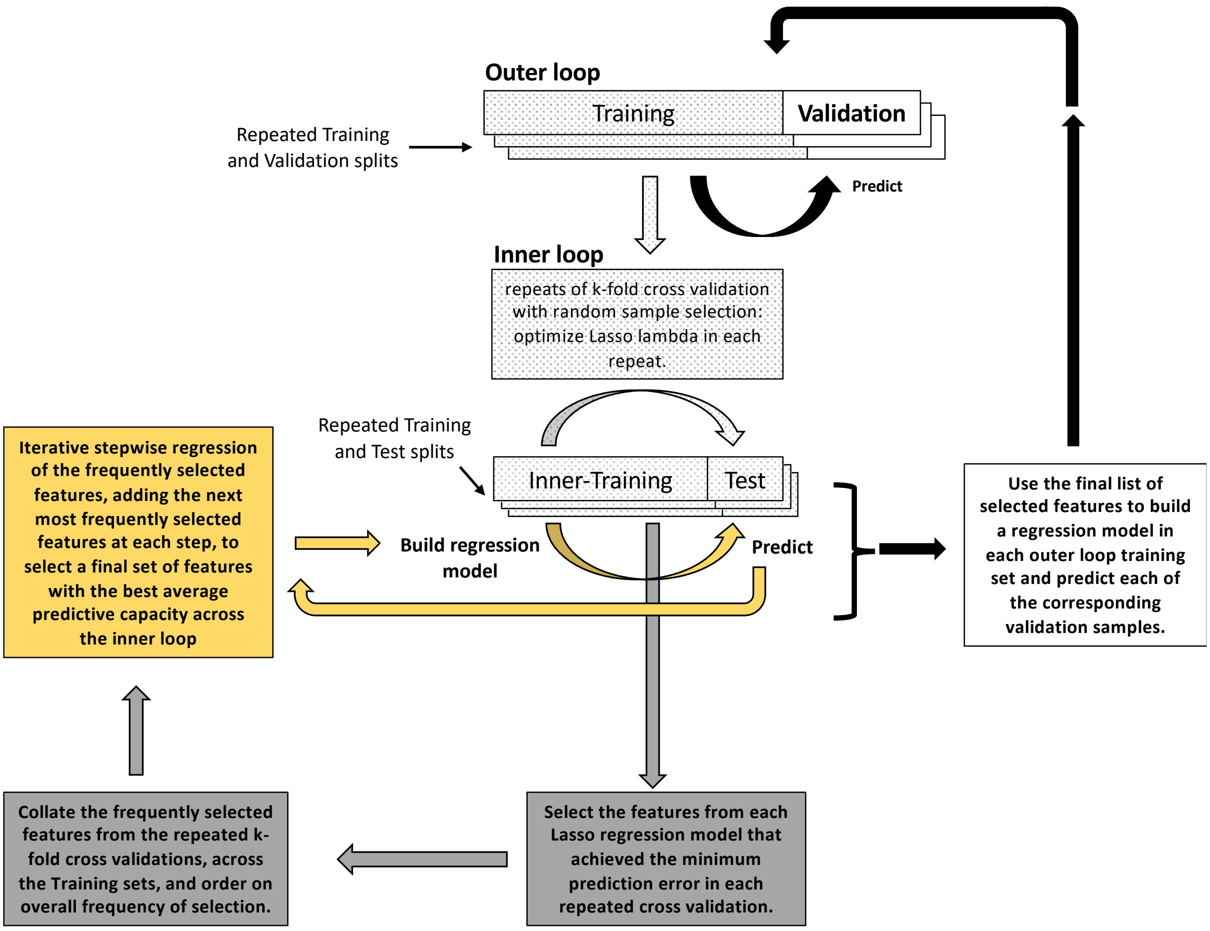

Supplementary Schema: Stable nested cross validation (StaVarSel) scheme: In the inner loop the level of regularisation (lambda) for the regression model was optimised via repeated 10-fold cross validation. The miR-ratios derived from applying lasso regression with the optimised lambda to each training set were collated, ranked according to frequency of selection, and then subjected to a form of stepwise forward regression to determine the optimum model with the least prediction error. The miR-ratios selected from the inner loop cross validation were used to build regression models in each training set, and these models were then used to make predictions for the held-out samples.
